# Optimal Duration of Umbilical Cord Clamping with Ventilation in a Preterm Asphyxiated Ovine Model

**DOI:** 10.3390/children12111462

**Published:** 2025-10-28

**Authors:** Mausma Bawa, Sylvia Gugino, Justin Helman, Nicole Bradley, Lori Nielsen, Arun Prasath, Clariss Blanco, Mary Divya Kasu, Hamza Abbasi, Munmun Rawat, Praveen Chandrasekharan

**Affiliations:** 1 Department of Pediatrics, State University of New York at Buffalo, Buffalo, NY 14203, USA; sfgugino@buffalo.edu (S.G.); jhelman@buffalo.edu (J.H.); nkbradle@buffalo.edu (N.B.); lnielsen@buffalo.edu (L.N.); hamzaabb@buffalo.edu (H.A.); munmunra@buffalo.edu (M.R.); 2 Division of Neonatology, Department of Pediatrics, University of Texas at Southwestern, Dallas, TX 75390, USA; arun.prasath@utsouthwestern.edu; 3 Division of Neonatology, Department of Pediatrics, NYC Health and Hospitals/Harlem, New York, NY 10037, USA; blancoc1@nychhc.org; 4 Division of Neonatology, University of Maryland Medical Center, Baltimore, MD 21201, USA; mkasu@som.umaryland.edu

**Keywords:** delayed cord clamping, non vigorous preterm neonate, neonatal resuscitation, neonatal transition, asphyxia, ventilation, oxygenation

## Abstract

**Highlights:**

**What are the main findings?**

- Delayed umbilical cord clamping with ventilation (DCCV) for 5 min significantly increased the proportion of preterm asphyxiated lambs achieving heart rate ≥ 100 bpm and oxygen saturation ≥80% within 5 min of resuscitation.
- DCCV for 5 min also improved pulmonary blood flow and gas exchange compared to shorter DCCV durations and early cord clamping with ventilation (ECCV).

**What is the implication of the main finding?**

- Initiating ventilation while maintaining an intact umbilical cord for 5 min may facilitate a smoother cardiopulmonary transition in preterm neonates.
- These findings highlight the potential physiological benefits of prolonged DCCV and warrant further validation in clinical studies.

**Abstract:**

**Background**: There is inadequate evidence to support recommendations for the delayed clamping of umbilical cords in preterm neonates who are born non-vigorous. **Objective**: In a preterm bradycardic ovine model, our objective was to compare the effects of early cord clamping with ventilation (ECCV) and various time periods of delayed cord clamping with ventilation (DCCV) at 1 min (DCCV1), 2 min (DCCV2), 3 min (DCCV3), 4 min (DCCV4), and 5 min (DCCV5). The primary composite outcome was (i) incidence of achieving a combined heart rate (HR) ≥ 100 bpm and preductal saturation (SpO_2_) ≥80% by 5 min, and (ii) time to attain this outcome. Secondary outcomes were to evaluate gas exchange/hemodynamics. **Methods:** 32 preterm lambs of 126–128-day gestational age were randomized to one of six groups: ECCV (*n* = 5), DCCV1 (*n* = 6), DCCV2 (*n* = 5), DCCV3 (*n* = 6), DCCV4 (*n* = 6), and DCCV5 (*n* = 4). Asphyxia was induced by umbilical cord occlusion to attain a HR ≤ 90 beats per minute (bpm). **Results**: All lambs in DCCV5 achieved a primary composite outcome by 5 min. The time taken to achieve the primary composite outcome in DCCV5 was significantly lower (*p* = 0.02). Partial pressure of arterial carbon dioxide (PaCO_2_) was significantly lower (*p* = 0.0001) in DCCV5. Peak pulmonary blood flow (PBF) was significantly higher (*p* = 0.0001) in DCCV5 while peak carotid blood flow (CBF) was highest in the ECCV (*p* < 0.0001) compared to other groups. **Conclusions**: In a preterm ovine model of asphyxia, resuscitation with an intact umbilical cord for 5 min increased the incidence and reduced the time to achieve the primary composite outcome, while also improving gas exchange by enhancing pulmonary blood flow, compared to shorter durations of DCCV and ECCV. These findings suggest that DCCV for 5 min may offer physiological advantages in the resuscitation of non-vigorous preterm neonates, warranting further investigation in clinical settings.

## 1. Introduction

Preterm birth, defined as delivery before 37 weeks of gestation, affects approximately 13.4 million infants annually worldwide [1]. Multiple governing bodies, including the International Liaison Committee on Resuscitation (ILCOR) [2] and the American College of Obstetricians and Gynecologists (ACOG) [3], recommend delayed cord clamping (DCC) for 30 to 60 s in vigorous term and preterm neonates.

The World Health Organization (WHO) [4] recommends a delay of 60–180 s before clamping the cord. More recently, updated guidelines by the American Heart Association and the American Academy of Pediatrics state that intact cord milking may be considered an alternative to early cord clamping in non-vigorous term and late preterm infants (35–42 weeks gestation) [5]. However, there remains a lack of consensus regarding optimal cord management in non-vigorous preterm neonates at birth, a group at particularly high risk.

DCC in preterm infants has been associated with benefits including reduced need for blood pressure support medications, decreased transfusions, and reduced hospital mortality [6,7,8]. The transition from fetal to neonatal circulation involves cessation of placental oxygen delivery, closure of fetal shunts, and a reduction in vascular resistance following pulmonary vasodilation. Establishing ventilation during DCC in preterm neonates may facilitate more effective cardiopulmonary transition [9,10,11]. Notably, many clinical trials have excluded neonates with perinatal compromise, particularly neonates who are pale, limp, or nonresponsive, despite their increased vulnerability and potential to benefit most from DCC.

The timing of umbilical cord clamping holds significant implications for the health and well-being of preterm neonates, specifically those requiring resuscitation in the delivery room. Recent meta-analyses demonstrate that prolonged cord clamping (≥120 s) reduces mortality before discharge in preterm infants [12]. Despite its simplicity and effectiveness, the optimal timing for cord clamping remains a subject of ongoing research.

Based on this background, our objective in this study was to compare the effects of early cord clamping with ventilation (ECCV) and various time periods of delayed cord clamping with ventilation at 1, 2, 3, 4 and 5 min, respectively (DCCV1, DCCV2, DCCV3, DCCV4 and DCCV5) using a preterm asphyxiated ovine model. Our primary composite outcome was (i) to achieve a combined HR ≥ 100 bpm & preductal SpO_2_ ≥ 80% by 5 min, and (ii) the time to achieve the combined outcome. Our secondary outcome was to measure the effect on gas exchange and hemodynamics.

## 2. Materials and Methods

The study was approved by the Institutional Animal Care and Use Committee (IACUC) at the State University of New York, Buffalo, NY, USA (PED 10085N). All experiments performed in this study adhered to the ARRIVE guidelines [13]. A total of 32 preterm time-dated ewes (125–127 days gestation, approximately equivalent to 28-week human neonates) were sourced from Maple Twig Farms, Meyersdale, PA, USA. These ewes did not receive antenatal steroids, to reflect the acute preterm birth scenarios when sufficient time for steroid administration is not available particularly in emergent deliveries due to perinatal asphyxia. After induction of anesthesia, the ewes were intubated using a 10.0 mm-cuffed endotracheal tube (ETT) and ventilated with a mixture of 100% oxygen and 2–3% isoflurane at a rate of 16 breaths per minute. Continuous monitoring of the ewes was conducted using a pulse oximeter and an end-tidal CO2 (EtCO_2_) monitor. Subsequently, a cesarean section was performed, and the lambs were partially exteriorized. They were intubated with a cuffed 4.5 mm ETT, and lung liquid was allowed to drain passively by gravity. To prevent air entry during gasping, the ETT was occluded prior to delivery. A jugular venous line was placed for access and blood draw. The right carotid artery was catheterized for pressure monitoring and for arterial blood gas draws. Blood flow transducers (Transonic, Ithaca, NY, USA) were placed to monitor the left common carotid blood flow, left pulmonary artery blood flow, and ductal flow. To ensure accurate preductal oxygen saturation (SpO_2_) readings, two sensors were placed: one on the tongue and one on the right upper limb (Nonin X-100 Sensmart; Plymouth, MN, USA). Additional probes were also placed on the umbilical artery and umbilical vein to monitor flows. The fetus was then completely delivered, placed on the ewe’s abdomen, and covered with warm towels and heat packs to maintain temperature, avoiding tension on the umbilical cord. Asphyxia was induced by umbilical cord occlusion until the HR was less than 90 bpm. We targeted a HR < 90 bpm to ensure that the preterm lambs had a consistent HR < 100 bpm at onset of resuscitation.

**Figure 1:**
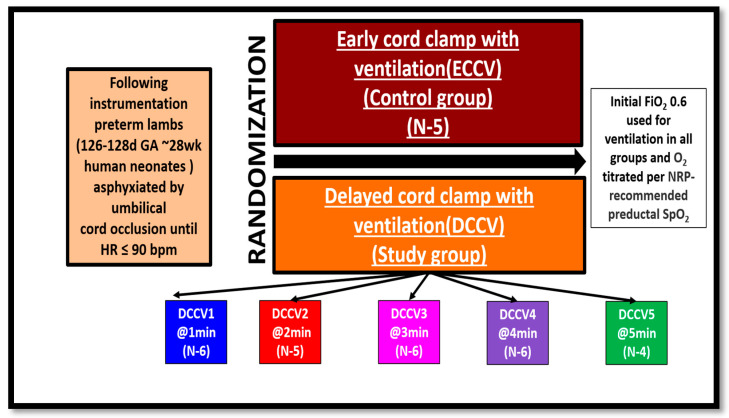
Design and Methodology.

*Group allocation and design*: Lambs were randomized to study groups using a computer-generated random sequence prior to delivery. Group allocation was concealed in sealed opaque envelopes. Due to the nature of the surgical and ventilatory interventions, blinding of the research team was not feasible. Once the target HR was achieved, the lambs were randomized to one of the six groups: (i) ECCV (control, current standard of care) (ii) DCCV1, (iii) DCCV2, (iv) DCCV3, (v) DCCV4, and (vi) DCCV5. In the ECCV (Early Cord Clamping with Ventilation) group, the umbilical cord was clamped immediately upon achieving the target HR, (HR < 90 bpm) followed by initiation of ventilation. In the DCCV1 group (Delayed Cord Clamping with Ventilation—1 min), the cord was released after asphyxia, (HR < 90 bpm) with ventilation initiated while the cord remained intact; and the cord was clamped at 1 min. The DCCV2, DCCV3, DCCV4, and DCCV5 groups followed the same protocol with cord clamping delayed until 2, 3, 4, and 5 min, respectively. This design allowed assessment of the effects of progressively prolonged ventilation with an intact umbilical cord (Figure 1).

Once the target HR < 90 bpm was achieved, the ETT occluder was removed, and positive pressure ventilation (PPV) was initiated. A T-piece device (Neo -Tee infant, Mercury Medical, Clearwater, FL, USA) with peak inspiratory pressure (PIP) of 35 cm H_2_O and positive end expiratory pressure (PEEP) of 5 cm H_2_O and respiratory rate (RR) of 40–60 breaths/min; targeting tidal volumes of 8–10 mL/kg was used for PPV delivery. We continuously measured end-tidal carbon dioxide, peak inspiratory pressure, positive end-expiratory pressure, respiratory rate, and tidal volume using a respiratory profile monitor (Respironics NM3, Philips, Murrysville, PA, USA).

The initial supplemental oxygen was set to 60% based on our previous study protocol in preterm lambs [14] and adjusted every minute based on Neonatal Resuscitation Program (NRP) SpO_2_ targets: If preductal SpO_2_ was outside the target range, the inspired O_2_ was adjusted every min as follows: (a) Adjusted by 10% if SpO_2_ less than 10% outside the range (e.g., target SpO_2_ 70%, previous minute SpO_2_ 60%, then increase O_2_ by 10%) or (b) Adjusted by 20% if SpO_2_ greater than 10% outside the range (e.g., target SpO_2_ 80%, previous minute SpO_2_ 50%, then increase O_2_ concentration by 20%). FiO_2_ was similarly reduced based on SpO_2_ exceeding the target.

Arterial blood gases were collected at baseline, just before ventilation at peak asphyxia, and then every minute for the first 10 min, followed by every 5 min until 30 min, and then every 15 min until 2 h post-delivery. Hemodynamic parameters, including left carotid, pulmonary artery, and systemic blood pressures, were continuously recorded using AcqKnowledge Acquisition and Analysis Software (BIOPAC Systems, Goleta, CA, USA, Version 5.0. 2.0). Lambs were given IV fluids (5 mL/kg/h) and sedation with IV fentanyl (0–2 ug/kg/h, titrated to effect) or IV diazepam (0.5 mg/kg; bolus as needed) if they demonstrated pain by toe pinch, movements, sustained increase in heart rate or blood pressure during the ventilation period. Lambs were humanely euthanized with an overdose of intravenous pentobarbital sodium (Fatal-Plus, Vortech Pharmaceuticals, Dearborn, MI, USA) at the conclusion of the experimental protocol.

### Data Collection and Statistical Analysis

**Figure 2:**
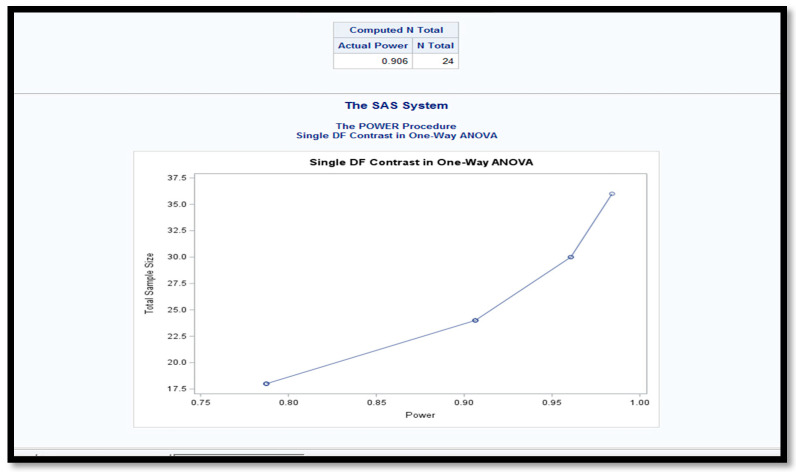
Sample size estimation was performed using one-way ANOVA in SAS 9.4. Single contrast coefficients were applied for ANOVA post hoc analysis, assuming an estimated group mean difference of 3 min and a standard deviation of 2 min. The x-axis shows the power and the y-axis the sample size. The advancing line graph shows an upward curve with 32 lambs showing a power of ~0.96.

Sample size estimation was performed using one-way ANOVA using the SAS 9.4 software (Cary, NC, USA). Single contrast coefficients were applied for ANOVA post hoc analysis, assuming an estimated group mean difference of 3 min and a standard deviation of 2 min. Although a total of 24 lambs were required to achieve a statistical power of 0.906 at an alpha level of 0.05, 32 preterm lambs were randomly assigned to the different groups which led to disparity in numbers in the groups (Figure 2).

Data distribution was evaluated using the Kolmogorov–Smirnov test. Demographic variables were summarized as frequencies and percentages, means ± standard deviations, or medians with interquartile ranges, as appropriate. Group comparisons were performed using Chi-square tests, unpaired *t*-tests, ANOVA, or their nonparametric equivalents.

Statistical analyses were conducted using IBM SPSS Statistics (version 31.0.1.0; IBM Corp., Armonk, NY, USA) and SAS software (version 9.4; SAS Institute Inc., Cary, NC, USA), with statistical significance defined as *p* < 0.05. All randomized lambs were included in the final analysis.

## 3. Results

In this randomized study, thirty-two preterm lambs with gestation ages of 125–127 days were randomly assigned into one of six groups: five in ECCV, six in DCCV1, five in DCCV2, six in DCCV3, six in DCCV4, and four in DCCV5. Table 1 presents the characteristics of all lambs. The groups exhibited similarity in gestational age, birth weight, sex distribution, heart rate, arterial pH, PaCO_2_, and PaO_2_ following asphyxiation before the initiation of the experimental protocol.

**Figure 3:**
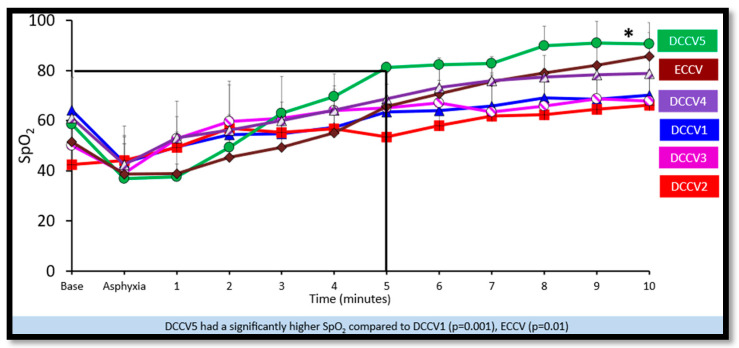
Preductal Saturations. The graph here shows the time in minutes during resuscitation along the X axis and the preductal saturations on the Y axis. *: DCCV5 had significantly higher preductal saturations compared to DCCV1 (*p* = 0.001), DCCV3 and ECCV (*p* = 0.01). The black line shows the DCCV5 group attained preductal saturation of 80% by 5 min.

**Figure 4:**
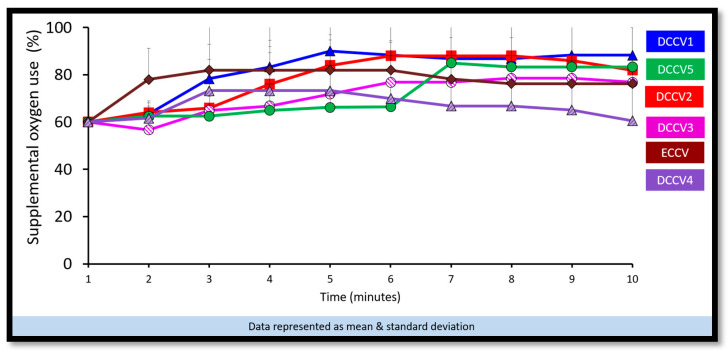
Supplemental oxygen use during the first ten minutes of resuscitation.

**Figure 5:**
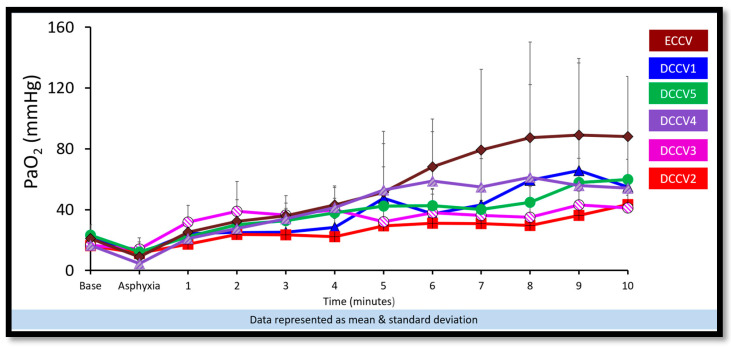
Partial pressure of oxygen in arterial blood. The graph here shows the time in minutes during resuscitation along the X axis and the arterial oxygen (PaO_2_) on the Y axis. There was no statistical difference between the partial pressure of oxygen in arterial blood.

**Figure 6:**
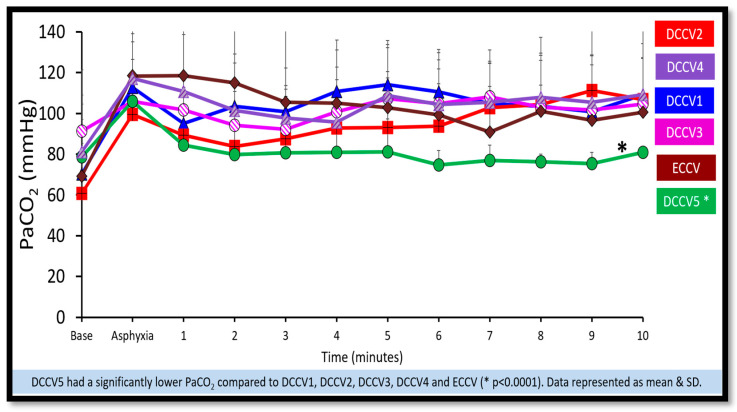
Partial pressure of carbon dioxide in arterial blood. The graph here shows the time in minutes during resuscitation along the X axis and the arterial carbon dioxide (PaCO_2_) on the Y axis. Arterial carbon dioxide was significantly lower (*p* = 0.0001) in DCCV5 compared to other groups.

**Figure 7:**
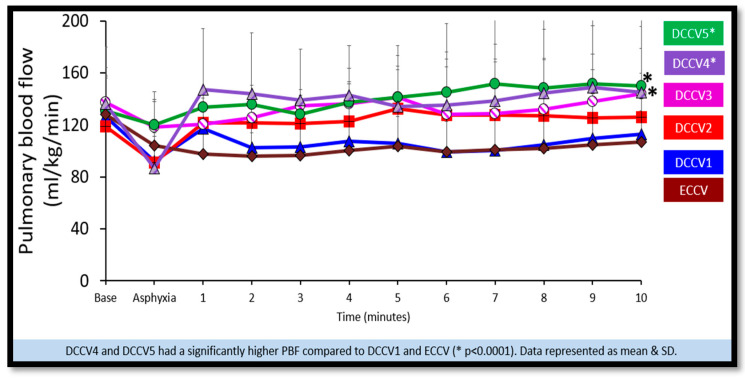
Pulmonary blood flow. The graph here shows the time in minutes during resuscitation along the X axis and the pulmonary blood flow (mL/kg/min) on the Y axis. The peak pulmonary blood flow (PBF) was significantly higher (*p* < 0.0001) in DCCV4 and DCCV5 compared to ECCV & DCCV1.

**Figure 8:**
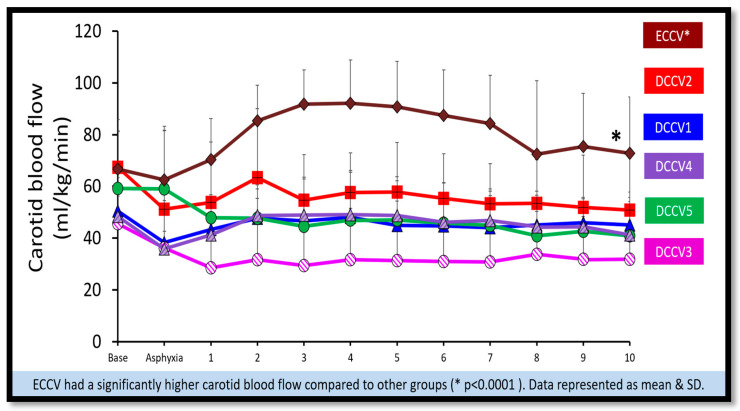
Carotid blood flow. The graph here shows the time in minutes during resuscitation along the X axis and the carotid blood flow (mL/kg/min) on the Y axis. ECCV had a significantly higher carotid blood flow compared to other groups (* *p* < 0.0001).

(i) Primary Composite Outcome
  (a) Incidence of achieving combined heart rate (HR) ≥ 100 bpm and preductal saturation (SpO_2_) ≥ 80% by 5 min: (Table 2)
    All lambs in DCCV5 (100%) achieved the primary composite outcome of a heart rate (HR) of ≥100 bpm and a preductal oxygen saturation (SpO_2_) level of ≥80% by 5 min. In ECCV and DCCV2, only 20% of lambs achieved this primary composite outcome, while DCCV1 and DCCV4 saw 16% success in attaining the criteria. DCCV3 had 33% of lambs meeting the composite outcome of HR ≥ 100 bpm and preductal SpO_2_ ≥ 80% by 5 min.
  (b) Time taken to achieve combined heart rate (HR) ≥ 100 bpm & preductal saturation (SpO_2_) ≥ 80% by 5 min: (Table 2)
    The time taken to achieve the primary outcome was significantly lower in DCCV5 (5 ± 1 min) compared to DCCV1 (13 ± 6 min) (*p* = 0.02—post hoc, ANOVA). With ECCV, it took 8 ± 3 min, DCCV2 required 10 ± 4 min, while DCCV3 took 11 ± 5 min. DCCV4 attained the primary outcome in 8 ± 2 min.
(ii) Secondary outcomes: Gas exchange and hemodynamics
  (a) Preductal saturations: As shown in Figure 3, DCCV5 had significantly higher preductal saturations compared to DCCV1, DCCV3 and ECCV. DCCV5 attained a preductal saturation of 80% in 5 min.
  (b) Supplemental oxygen use: The supplemental oxygen used during the first ten minutes is shown in Figure 4. An initial supplemental oxygen of 60% was used in all groups in our study. There was no statistical difference between the groups’ supplemental O_2_ exposure. Oxygen was titrated according to the NRP recommended preductal saturation targets.
  (c) Partial pressure of oxygen in arterial blood (PaO_2_): Figure 5 shows the partial pressure of oxygen in arterial blood in the first ten minutes. There was no statistical difference in PaO_2_ between the groups. Despite starting at a higher supplemental oxygen level of 60% in all the groups, none of the groups exceeded mean PaO_2_ values of 100 mmHg within the initial ten minutes while weaning FiO_2_ per NRP recommendations.
  (d) Partial pressure of carbon dioxide in arterial blood (PaCO_2_): DCCV5 had significantly lower carbon dioxide in arterial blood (PaCO_2_) compared to other groups (*p* = 0.0001) as shown in Figure 6.
(iii) Hemodynamics:
  (a) Pulmonary blood flow: The peak pulmonary blood flow in the first ten minutes is shown in Figure 7. DCCV4 and DCCV5 had a significantly higher peak pulmonary blood flow compared to ECCV and DCCV1 (*p* < 0.0001). The pulmonary blood flow was lowest in the ECCV group.
  (b) Carotid blood flow: The peak carotid blood flow during the first ten minutes is shown in Figure 8. The highest peak carotid blood flow was seen in the ECCV group and was statistically different (*p* = < 0.0001) compared to other groups.

## 4. Discussion

In its 8th edition, the Textbook of Neonatal Resuscitation included umbilical cord management as one of the four critical pre-birth questions, highlighting its growing clinical importance [5]. While extensive meta-analyses support the benefits of delayed cord clamping (DCC), the optimal timing of delayed clamping particularly in non-vigorous or asphyxiated preterm infant remains uncertain. The current study contributes to this knowledge gap by evaluating different durations of delayed cord clamping with ventilation (DCCV) in an asphyxiated preterm ovine model.

A recent individual patient data meta-analysis by Seidler et al. evaluated 48 randomized trials including 6367 preterm infants and demonstrated that deferred cord clamping compared to early clamping reduced death before discharge with an odds ratio of 0.68 [95% CI 0.51–0.91] [15]. Another systematic review by Liyanage et al. of 44 clinical guidelines revealed broad agreement on the benefits of DCC in preterm infants, although it noted a lack of consistency regarding the recommended duration of the delay [16]. Seidler et al. also compared the short, medium and long delays of cord clamping with umbilical cord milking and analyzed participant data from 47 trials involving 6094 infants concluding that long deferral (≥120 s) had a 91% probability of being the most effective strategy for reducing mortality, whereas immediate clamping had a 53% probability of being the least effective [12].

Current evidence on optimal cord management in non-vigorous preterm neonates remains limited. A recent systematic review and network meta-analysis evaluating cord management strategies in non-vigorous newborns suggested that both intact cord resuscitation (ICR) and UCM may be safe and potentially effective alternatives to ECC. Reported benefits included higher 5 min Apgar scores and improved hematological parameters, such as enhanced iron stores [17].

A key animal-model study demonstrated that preterm lambs randomized to cord clamping after initiation of ventilation (Vent 1st) had significantly improved cardiovascular stability compared to lambs in which the cord was clamped before ventilation. Specifically, lambs in the clamp-first group exhibited a ~40% reduction in HR and large drop in right ventricular output, whereas the ventilation-first group maintained stable flows and pressures [8]. Likewise, Graeme R. Polglase and colleagues found that initiating ventilation before cord clamping improved systemic and cerebral oxygenation in preterm lambs [18]. Our results extend these physiological insights by showing that in an asphyxiated preterm model, DCCV for 5 min (DCCV5) led to all lambs achieving the primary composite outcome (HR ≥ 100 bpm and preductal SpO_2_ ≥ 80%) by 5 ± 1 min, compared to 13 ± 6 min in the DCCV1 group. These data suggest that prolonged DCC with ventilation may further enhance transition outcomes in compromised preterm births.

Clinical studies also support these findings. Padilla-Sánchez et al. reported that term infants with delayed cord clamping had higher SpO_2_ and HR in the first five minutes post-birth compared to those with immediate clamping [19]. Similarly, the Nepcord III randomized controlled trial (RCT) involving 1560 women demonstrated that intact cord resuscitation resulted in better oxygenation and earlier initiation of breathing in late preterm and term neonates requiring resuscitation [20]. In another recent systematic review and meta-analysis, no significant differences were observed in delivery room parameters, in-hospital mortality, or early complications of prematurity between infants who received resuscitation with an intact cord and those who underwent immediate cord clamping. However, the intact cord group demonstrated a higher SpO_2_ at 5 min after birth (mean difference 6.67%, 95% CI [−1.16%, 14.50%]), indicating potential benefits in early optimized oxygenation [21].

Ventilation of the lungs is the cornerstone of effective neonatal resuscitation [5]. Extreme preterm infants, who are often surfactant-deficient, frequently require respiratory support. In non-vigorous infants, additional factors such as acidosis and hypoxia can impede normal cardiopulmonary transition. In our study, the DCCV5 group showed higher preductal SpO_2_ levels at five minutes, with no significant difference in supplemental oxygen exposure between groups. Furthermore, lambs in the DCCV5 group had significantly lower arterial carbon dioxide levels and reduced carotid blood flow, which may confer protection against IVH, providing additional support for the benefits of prolonged DCC with ventilation (Figure 6 and Figure 8).

Is a supplemental oxygen of 21–30% adequate for transitioning preterm non vigorous neonates? The current neonatal resuscitation guidelines, which were largely informed by studies in spontaneously breathing infants, recommend initiating resuscitation in preterm infants with 21–30% oxygen [22]. However, emerging evidence suggests that this may not be adequate in non-vigorous preterm neonates. A meta-analysis by Oei et al. of eight RCTs involving infants <32 weeks’ gestation reported a threefold increase in the risk of intraventricular hemorrhage and death in infants who experienced bradycardia and oxygen saturations <80% within five minutes of birth [23]. Similarly, Dekker et al. conducted a small RCT in infants <30 weeks’ gestation and found that initiating resuscitation with 100% oxygen, followed by titration, improved breathing effort and oxygenation without increasing the risk of hyperoxia [24]. In a 2021 study by Chandrasekharan et al., asphyxiated preterm lambs were randomized to either immediate cord clamping or delayed cord clamping (DCC) starting resuscitation with either 0.3 or 0.6 FiO_2_. Lambs in the DCC group with higher initial oxygen achieved target SpO_2_ earlier, required lower FiO_2_, and experienced a reduced oxygen load. This concept of “differential oxygenation,” where the placenta serves as an additional regulator of PaO_2_ during resuscitation, may help protect against the potential harms of higher initial FiO_2_ exposure [14]. A recent individual participant data network meta-analysis evaluated the impact of initial oxygen concentration during delivery room resuscitation in infants born <32 weeks’ gestation. The analysis, which included 1055 infants from 12 randomized trials, found that resuscitation with high FiO_2_ (≥0.90) was associated with a significantly lower risk of mortality compared to both low (≤0.3) and intermediate (0.5–0.65) FiO_2_, although the certainty of evidence was low to very low [25]. Three recent multicenter randomized clinical trials have also evaluated the effects of providing respiratory support during delayed cord clamping in very preterm infants. The ABC3 trial compared physiological-based cord clamping to time-based clamping and found no significant difference in intact survival, although fewer transfusions and infections were observed in the physiological group [26]. Pratesi et al. compared resuscitation with intact placental circulation to umbilical cord milking and similarly reported no significant improvement in the composite outcome of death, severe IVH, or BPD [27]. In the VentFirst trial, Fairchild et al., investigated providing ventilatory assistance for up to 2 min before cord clamping and found no reduction in early death or IVH, although subgroup analysis suggested possible benefit in non-breathing infants [28]. Notably, all three trials used relatively low initial oxygen concentrations—30% [27,28] to 45% [26] during resuscitation. Collectively, these studies suggest that while intact cord resuscitation is feasible and safe, using low FiO_2_ may limit its effectiveness in promoting pulmonary transition and improving major clinical outcomes.

The recently published DOXIE randomized controlled trial by Katheria et al. further supports this concept. In this study, administering 100% oxygen during DCC in extremely preterm infants significantly improved early oxygenation—69% of infants reached SpO_2_ ≥ 80% by five minutes, compared to 39% with 30% oxygen—without an associated increase in morbidity, suggesting that higher initial FiO_2_ may be both safe and beneficial in this setting [29]. In our previous study, we found that 30% supplemental O_2_ failed to achieve preductal SpO_2_ of 80% by five minutes in any preterm lamb [30]. In the current study, we used 60% supplemental O_2_ for initial resuscitation in all groups. No group exceeded PaO_2_ levels of 100 mmHg within the first 10 min, suggesting that high supplemental oxygen up to 60% may be safe and more effective in this context especially when the NRP suggested weaning targets are used.

Our findings suggest that DCCV for up to 5 min along with higher FiO_2_ offers a physiological advantage in asphyxiated preterm neonates. This strategy may facilitate a smoother cardiopulmonary transition compared to early cord clamping with immediate ventilation. We hypothesize that extending DCCV beyond 30–60 s could be beneficial for non-vigorous preterm neonates requiring resuscitation.

### Limitations

This study has several limitations. The ovine model used involves cesarean delivery under general anesthesia, absent uterine contractions, and species-specific anatomical differences—such as a shorter umbilical cord with two arteries and two veins. Placental separation dynamics also differ. Nonetheless, fetal lambs possess pulmonary physiology that is comparable to that of human neonates, and this model remains well-established for translational research in neonatology. Another limitation is the small sample size, as the potential benefits of DCCV5 in this study are based on findings from only four animals, which were randomized into different groups. We have previously reported that ventilation with an intact cord for 5 min in a preterm asphyxiated model, led to improved ventilation and pulmonary blood flow [10,14]. Our study has certain strengths. To our knowledge, this is the first study to examine various durations of DCCV in a bradycardic preterm ovine model. Additionally, our approach using higher initial FiO_2_ with titration based on preductal SpO_2_ may inform future clinical studies targeting this vulnerable population.

## 5. Conclusions

In a preterm ovine model of asphyxia due to bradycardia at birth, delayed cord clamping with ventilation for 5 min increased incidence to attain primary outcome of HR ≥ 100 bpm & preductal SpO_2_ ≥ 80% by 5 min, decreased the time to achieve the primary outcome and improved gas exchange by increasing pulmonary blood flow. Research involving optimal time for delayed cord clamping and ventilation using larger sample size translational models and clinical studies in preterm neonates requiring resuscitation is urgently needed to inform future guidelines in this vulnerable population.

## Figures and Tables

**Table 1:** Baseline Characteristics of the Lambs.

Characteristic	ECCV (N-5)	DCCV1 (N-6)	DCCV2 (N-5)	DCCV3 (N-6)	DCCV4 (N-6)	DCCV5 (N-4)
**Gestational age (days)**	128 ± 0.84	126 ± 0.47	126 ± 0.97	126 ± 1.5	126 ± 0.47	127 ± 0.52
**Female (N)**	3	5	2	3	3	4
**Birth weight (kg)**	3.3 ± 0.70	2.9 ± 0.35	2.9 ± 0.42	3.0 ± 0.30	3.16 ± 0.45	3.3 ± 0.63
**Heart rate at asphyxia (bpm)**	86 ± 10	80 ± 9	87 ± 2	86 ± 4	85 ± 13	88 ± 8
**pH before resuscitation**	6.91 ± 0.04	6.98 ± 0.07	7.07 ± 0.07	7.00 ± 0.09	6.96 ± 0.08	6.98 ± 0.06
**PaCO_2_ before resuscitation (mmHg)**	119 ± 31	113 ± 22	100 ± 2	106 ± 21	117 ± 22	106 ± 11
**PaO_2_ before resuscitation (mmHg)**	9 ± 6	11 ± 6	12 ± 6	14 ± 5	8 ± 3	12 ± 9

Data presented as numbers or as average and standard deviation.

**Table 2:** Primary outcome: (a) Incidence of achieving a combined HR > 100 bpm and preductal SpO_2_ ≥ 80% by 5 min (b) Time taken to achieve the primary outcome.

Parameter	Control (ECCV) (N-5)	STUDY (DCCV)				
		DCCV1 (N-6)	DCCV2 (N-5)	DCCV3 (N-6)	DCCV4 (N-6)	DCCV5 * (N-4)
Combined HR ≥ 100 bpm & preductal SpO_2_ ≥ 80% by 5 min N (%)	1/5 (20%)	1/6 (16%)	1/5 (20%)	2/6 (33%)	1/6 (16%)	4/4 (100%)
Time to achieve primary outcome (minutes)	8 ± 3	13 ± 6	10 ± 4	11 ± 5	8 ± 2	5 ± 1

Data represented N (%); mean & standard deviation. * *p* = 0.02.

## Data Availability

The original contributions presented in this study are included in the article. Further inquiries can be directed to the corresponding author.

## Abbreviations

The following abbreviations are used in this manuscript:

AAP-NRP	American Academy of Pediatrics Neonatal Resuscitation Program
ACOG	American College of Obstetricians and Gynecologists
DCC	Delayed cord clamping
DCCV	Delayed cord clamping with ventilation
ECC	Early cord clamping
ECCV	Early cord clamping with ventilation
ECG	Electrocardiogram
ETT	Endotracheal tube
ETCO_2_	End- tidal Carbon dioxide
FiO_2_	Fraction of inspired oxygen
IACUC	Institutional Animal Care and Use Committee
PPV	Positive pressure ventilation
PaCO_2_	Partial pressure of carbon dioxide in arterial blood
PaO_2_	Partial pressure of oxygen in arterial blood
NRP	Neonatal Resuscitation Program
SpO_2_	Preductal oxygen saturation
WHO	World Health Organization
